# Quantitative assessment of renal and perirenal adipose tissue distribution at 5 T: a feasibility study

**DOI:** 10.1186/s12880-025-02136-8

**Published:** 2025-12-29

**Authors:** Yichao Xu, Zhenxing Jiang, Runyu Tang, Shaofeng Duan, Jinggang Zhang, Tingting Zha, Wei Xing

**Affiliations:** 1 Department of Radiology, The Third Affiliated Hospital of Soochow University, 185 Juqian Street, Changzhou, China; 2 Collaborative Innovation Department, Shanghai United Imaging Healthcare Co., Ltd., Shanghai, China

**Keywords:** Magnetic resonance imaging, Ultra-high field, Proton density fat fraction, Renal fat, Perirenal fat, Adipose tissue

## Abstract

**Purpose:**

To evaluate the feasibility and accuracy of Fat Analysis Calculation Technique (FACT), a multi-echo Dixon-like sequence, for quantifying renal and perirenal adipose distribution at 5 T.

**Methods:**

Accuracy of FACT-based Proton density fat fraction (FACT-PDFF) was assessed by comparing with magnetic resonance spectroscopy-based PDFF (MRS-PDFF) in phantom study. In vivo FACT images from 24 volunteers (13 males and 11 females) without kidney-related diseases were acquired at 5 T and evaluated independently by two readers. Repeatability of FACT-PDFF was assessed through three consecutive scans. Spearman correlation examined associations between averaged FACT-PDFF and clinical characteristics. Linear regression, intraclass correlation coefficients (ICCs), and Bland-Altman plots assessed consistency and deviations between fat quantification methods and field strengths. The Wilcoxon signed-rank test compared image quality scores between radiologists. The paired t test compared FACT-PDFF differences across all regions of interest between bilateral kidneys and between renal cortex and medulla. Analysis of covariance compared gender-related renal fat differences.

**Results:**

In phantom study, FACT-PDFF showed excellent agreement with MRS-PDFF at both fields (ICCs ≥ 0.995). Linear regression revealed strong correlations (R² ≥ 0.998), and Bland-Altman plots indicated minimal bias. In clinical study, FACT images achieved high quality. Repeatability was excellent (ICCs: 0.837–0.991; CVs: 0.78–4.49%). Significant PDFF differences existed between bilateral kidneys (cortex/medulla: *P* < 0.001; sinus fat: *P* = 0.003), cortex vs. medulla (*P* < 0.001), and genders (right cortex, left medulla, and left perirenal fat: *P* ≤ 0.044). PDFF was correlated positively with age, weight, body mass index, waist/hip circumference, and waist-to-height ratio (*r* = 0.412–0.797, *P* ≤ 0.046).

**Conclusions:**

FACT at 5 T reliably quantifies renal and perirenal adipose distribution. It can offer a non-invasive alternative to biopsy, and may facilitate the understanding of renal adipose distribution and its clinical associations.

**Supplementary Information:**

The online version contains supplementary material available at 10.1186/s12880-025-02136-8.

## Introduction

In recent years, kidney-related diseases, including chronic kidney disease, diabetes-related nephropathy, and obesity-induced renal dysfunction, have emerged as a pressing global health issue [1–4]. These conditions not only severely deteriorate the quality of life of affected patients but also impose a substantial economic burden on healthcare systems worldwide. Despite substantial progress in medical research and clinical practice, early diagnosis and effective management of kidney-related diseases remain challenging.

Alterations in renal and perirenal fat content have been recognized as crucial factors influencing renal function. The underlying mechanism is likely associated with the excessive intracellular accumulation of fatty acids and triglycerides in the renal parenchyma. This excessive accumulation triggers a cascade of renal chronic cellular dysfunction and tissue damage, such as glomerulosclerosis, tubular injury, and interstitial fibrosis. These injuries, in turn, may ultimately lead to a loss of renal function [5–7]. Thus, accurately assessing renal and perirenal fat content is of utmost importance for understanding the pathogenesis and progression of kidney diseases.

Currently, pathological biopsy is regarded as the gold standard for evaluating changes in renal and perirenal fat content. However, its application is severely restricted due to inherent surgical risks and ethical considerations [8]. To overcome these limitations, non-invasive imaging techniques such as Dixon imaging and magnetic resonance spectroscopy (MRS) have emerged as promising alternatives. Dixon imaging is based on chemical shift encoded (CSE) magnetic resonance imaging (MRI), which relies on water-fat separation and assesses target region by measuring the proton density fat fraction (PDFF) [9]. In a wide spectrum of renal diseases, Dixon and Dixon-like imaging have reliably measured renal PDFF, which exhibited as an early biomarker for renal dysfunction [10–13]. Compared to conventional Dixon imaging, the Fat Analysis and Calculation Technique (FACT) utilizes a six-peak fat fitting model accounting for the complex spectral components of human fat, incorporates T2* correction algorithms to mitigate signal decay effects, and enables single-breath-hold acquisition to minimize motion artifacts, which has been extensively applied in the study of hepatic diseases and musculoskeletal system [14–17]. MRS still remains the gold standard for quantitative MRI assessments. Nevertheless, given the potential of FACT for fat quantification and the growing interest in 5 T MRI, it is important to explore its application in assessing renal and perirenal adipose tissue distribution. Compared to 3 T, chemical shifts between H protons in water and fat is amplified at 5 T, which potentially enhances the accuracy of fat quantification in renal and perirenal tissues.

Ultra-high field MRI (≥ 5 T) confers advantages for renal imaging, including superior signal-to-noise ratio (SNR), enhanced spatial resolution, and amplified water-fat chemical shift contrast. However, its clinical translation in renal imaging is hindered by organ-specific and technical challenges (e.g. exacerbated susceptibility artifacts, motion artifact vulnerability, amplified chemical shift edge artifacts). The most significant challenge lies in the inhomogeneity of the radio frequency (RF) transmit magnetic field (B₁⁺), as the wavelength of the RF field propagating within the human body is comparable to the body size. To mitigate this issue and improve B₁⁺ homogeneity, parallel transmission (pTx) technology equipped with an 8-channel RF power amplifier was implemented on the 5 T magnetic resonance imaging (MRI) system, which enabled the acquisition of high-quality renal MR images. Even so, the accuracy of fat quantification using 5 T awaits confirmation through additional studies.

Our present study aimed to evaluate the effectiveness of the FACT in quantifying renal and perirenal adipose tissue distribution in 5 T MRI. Through this effort, we aimed to provide novel ideas and methods for the diagnosis and treatment of renal diseases, contributing significantly to the progress of both basic scientific research and clinical practice.

## Methods

### Phantom study

A universal fat-water phantom was constructed containing 7 cylinders with a range of the volume fraction of the fat (0%, 10%, 20%, 30%, 40%, 50%, and 100%) to validate the accuracy and reproducibility of 5 T FACT-PDFF. The detailed phantom construction can be referred to the supplementary materials. Before scanning, the phantom was submerged in tap water at 35 °C for 10 min to stabilize and mimic the human body temperature. During the scanning process, the phantom remained submerged in water.

MRS and FACT were carried out on both 3 T system (uMR 790, United Imaging Healthcare) with a 24-channel head/neck coil and 5 T system (uMR Jupiter, United Imaging Healthcare) with a 48-channel head/neck coil. Detailed imaging parameters are shown in Table 1.

**Table 1:** Scanning parameters for MRS and FACT imaging in Phantom study

Parameters	MRS		FACT	
Field strength	3 T	5 T	3 T	5 T
Pulse sequence	Multi-echo single-voxel stimulated echo acquisition mode spectroscopy		Single-shot multi-echo gradient-recalled echo sequence	
First/Δ TE (msec)	12/12	10/5	1.47/1.60	0.97/0.97
Number of echoes	5	5	6	6
TR (msec)	2800	2800	11.27	7.45
Flip angle (degree)	90	90	3	3
VOI (mm^3^)	15 × 15 × 15	15 × 15 × 15	n.a.	n.a.
FOV (mm^2^)	n.a.	n.a.	384 × 384	384 × 384
Matrix	n.a.	n.a.	192 × 192	192 × 192
Slice thickness (mm)	n.a.	n.a.	2	2
Scan time (sec)	16.8	14.0	34.6	27.5

MRS, magnetic resonance spectroscopy; FACT, Fat Analysis and Calculation Technique; TE, echo time; TR, repetition time; VOI, volume-of-interest; FOV, field-of-view; n.a., not applicable

The MRS sequence employed the Stimulated Echo Acquisition Mode (STEAM) to continuously acquire data from a single voxel of each phantom tube. The STEAM method instead of point resolved spectroscopy (PRESS) was applied because PRESS might overestimate PDFF due to J coupling effects [18]. Postprocessing of MRS data at 3 T and 5 T was carried out using the workstation (uMR-WS, United Imaging Healthcare) by the in-built algorithm. A six-peak fitting model was applied to quantify the total fat content [19]. The methylene peak signal at 1.3 ppm, a prominent fat-associated signal, constitutes approximately 70% of the total fat content. T2 correction was achieved by fitting a single exponential curve to the fat and water peaks with multiple TE values [14]. The MRS-PDFF was determined via calculating the area under the fat and water peaks at a theoretical TE of 0 [20].

**Fig. 1:**
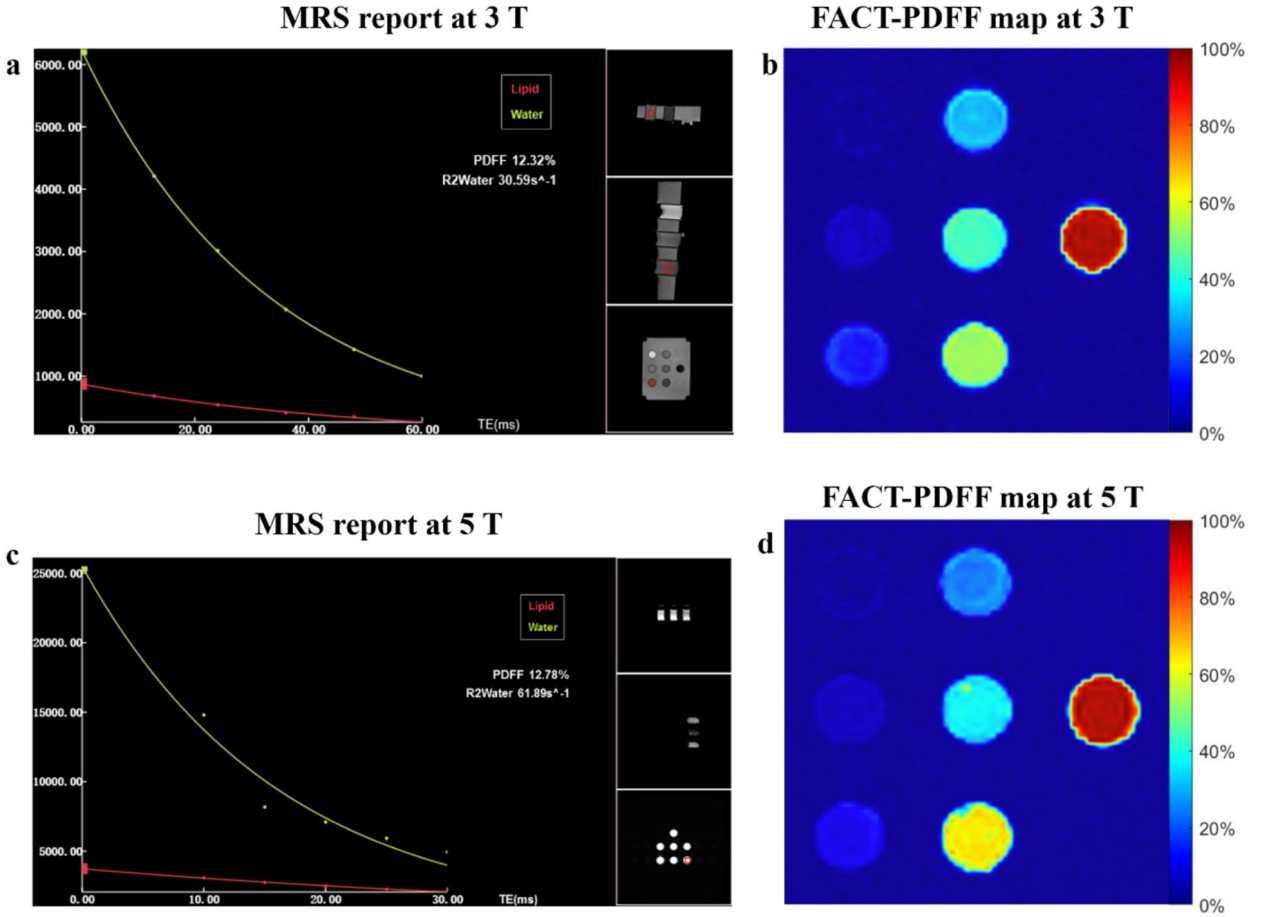
Representative phantom MRS-PDFF reports and FACT-PDFF maps at 3 T and 5 T. The MRS-PDFF of the VOI of a phantom tube was 12.32% at 3 T (**a**), and 12.78% at 5 T (**c**). FACT-PDFF maps of the phantom at 3 T (**c**) and 5 T (**d**). MRS, magnetic resonance spectroscopy; FACT, Fat Analysis and Calculation Technique; PDFF, proton density fat fraction; VOI, volume-of-interest

The FACT imaging used a 3D single-shot multi-echo gradient-recalled echo sequence. The FACT-PDFF map was also provided by inline reconstruction using a six-peak fat fitting model [21]. Representative MRS reports and FACT-PDFF maps of the phantom are shown in Fig. 1.

### Subjects

This prospective investigation was approved by the regional ethics committee. Before any of the volunteers were examined without kidney-related diseases, they all provided written informed consent. From May 2024 to December 2024, a total of 26 volunteers underwent renal magnetic resonance examinations. Two volunteers were excluded owing to their inability to access the scanning area as a result of over-obesity. In total, 24 volunteers were enrolled in this study.

### Clinical scan protocol

All MRI examinations were performed using the same 5 T scanner used in phantom study with a 24-channel body coil. The single breath-hold FACT sequence was acquired by the same sequence in phantom study with the following parameters: repetition time (TR), 7.65 ms; number of echoes, 6; first echo time (TE), 1.20 ms; ΔTE, 0.97 ms; flip angle, 3°; slice thickness, 4 mm; field-of-view (FOV), 240 × 288 mm^2^; matrix size, 160 × 192; acquisition time, 18.5 ms.

To evaluate the repeatability of FACT sequence, all participants underwent three consecutive scans without repositioning between any scan sessions. Volunteers were given a 40-second break during the scan interval.

### Image analysis

Two radiologists, with 5 and 10 years of experience in abdominal MRI diagnosis respectively, independently evaluated the in vivo images using a 5-point scoring system. The radiologists were blind to the clinical information and images were arranged in a random order. The scoring criteria for overall image quality were as follows: 1 = Nondiagnostic - unable to visualize the structure; 2 = Limited - able to see the structure but unable to evaluate; 3 = Diagnostic - able to evaluate the structure with some confidence; 4 = Good - able to evaluate the structure with high confidence; 5 = Excellent - the best quality of delineation.

FACT-PDFF maps were generated through the inline reconstruction of the scanner subsequent to the FACT examination. The two radiologists who were blind to participant information performed region-of-interest (ROI) measurement on the workstation (uMR-WS, United Imaging Healthcare) under the inspection of a third abdominal radiologist (with 15 years of experience in abdominal MRI). When disagreement occurred, a consensus was reached through discussion. For phantom imaging, square ROIs were carefully drawn and matched with the center slice of the imaging volume of the MRS sequence. For in vivo imaging, three central slices were selected through each side of the kidney. The radiologist manually delineated eight ROIs within the cortex, medulla, renal sinus fat, and perirenal fat regions of the bilateral kidneys on each slice. During this process, anatomical structures such as blood vessels, ureters, and renal edges were avoided, while covering the corresponding tissue area as comprehensively as possible. Then, the values of the three slices were averaged to produce the FACT-PDFF for each ROI.

### Statistical analysis

Continuous variables are presented as means ± standard deviations, and categorical variables are reported as counts and percentages. In phantom study, linear regression analysis, intraclass correlation coefficients (ICCs), and Bland-Altman plots were performed to evaluate the consistency and deviation between different fat quantification methods and field strengths.

In volunteer study, the normality of continuous data was assessed using the Kolmogorov-Smirnov test (Table S1). The Wilcoxon signed-rank test was used to compare the overall image quality scores between the two radiologists. The paired *t* test was used to compare the FACT-PDFF difference across all ROIs between bilateral kidneys as well as FACT-PDFF between cortex and medulla. To eliminate the influence of other clinical features on fat content, gender differences in renal fat were compared using analysis of covariance (ANCOVA). All collected clinical features except gender were set as covariates (Table 2). The repeatability and variability of FACT-PDFF obtained from multiple scans were assessed by ICCs and coefficients of variation (CVs). Both ICCs and CVs are reported with 95% confidence intervals (CIs). The ICC was calculated using a two-way and single-rater model, and was classified as: 0–0.39, poor; 0.40–0.59, fair; 0.60–0.74, good; and 0.75–1.0, excellent [22]. The within-subject CV was calculated as follows:

1 $$\:\begin{array}{c}CV\left(\mathrm{P}\mathrm{D}\mathrm{F}\mathrm{F}\right)=\frac{SD\left(PDFF\right)}{mean\left(PDFF\right)}\end{array}$$

CVs less than 15%, between 15% and 35%, and greater than 35% were considered small, moderate, and large variability, respectively.

As part of the continuous data were non-normal, averaged FACT-PDFF values from the three scanning sessions were correlated with clinical characteristics using Spearman correlation.

Data organization and statistical analysis were performed using SPSS version 23 software (IBM Corp., Armonk, NY). MedCalc (Version 20.0.4, Ostend, Belgium) was used to create Bland-Altman plots and calculate post-hoc power (Table S2). A two-sided *P* value < 0.05 was considered statistically significant.

**Table 2:** Characteristics of participants

Clinical features	Number
No. of subjects	24
Age (years)	31.5 ± 8.8
Sex	
Male	13 (54.2%)
Female	11 (45.8%)
Height (cm)	168.3 ± 8.5
Weight (kg)	64.7 ± 11.7
Waist (cm)	84.2 ± 11.1
Hip (cm)	97.0 ± 6.3
BMI (kg/m^2^)	22.8 ± 3.1
Waist-to-hip ratio	0.87 ± 0.08
Waist-to-height ratio	0.50 ± 0.06

BMI, body mass index

## Results

### Clinical features

A total of 24 volunteers were enrolled in this study. Characteristics of the study group are listed in Table 2. The cohort had a mean age of 31.5 ± 8.8 years, with a near-balanced sex distribution (13 males [54.2%] and 11 females [45.8%]). Characteristics of measurements indicated a normal body mass index (BMI) of 22.8 ± 3.1 kg/m², alongside additional body composition indices: mean height (168.3 ± 8.5 cm), weight (64.7 ± 11.7 kg), waist circumference (84.2 ± 11.1 cm), hip circumference (97.0 ± 6.3 cm), waist-to-hip ratio (0.87 ± 0.08), and waist-to-height ratio (0.50 ± 0.06).

### Phantom study

**Fig. 2:**
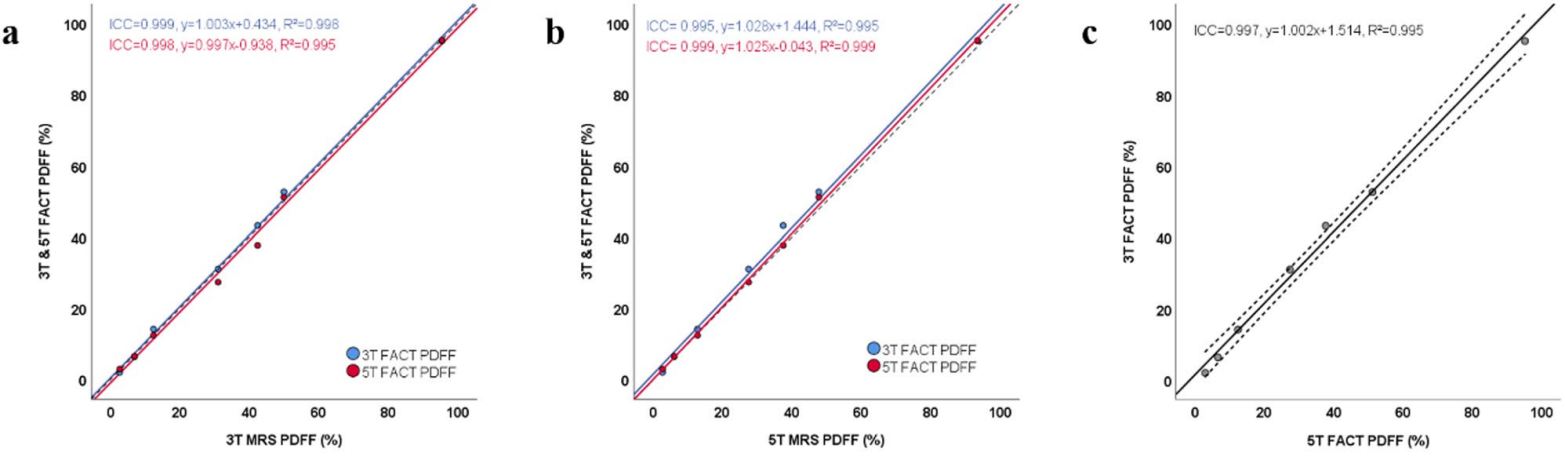
The consistency and linear regression analysis for FACT and MRS in the phantom. (**a**) Scatter plots of linear regression between 3 T, 5 T FACT-PDFF and 3 T MRS-PDFF. (**b**) Scatter plots of linear regression between 3 T, 5 T FACT-PDFF and 5 T MRS-PDFF. (**c**) Scatter plot of linear regression between 3 T and 5 T FACT-PDFF. FACT, Fat Analysis and Calculation Technique; MRS, magnetic resonance spectroscopy; PDFF, proton density fat fraction

In phantom study, the ICCs (0.995–0.999, *P* < 0.001 for all) for FACT-PDFF at different field strengths, as well as FACT-PDFF and MRS-PDFF at both 3 T and 5 T were in excellent agreement (Fig. 2).

**Fig. 3:**
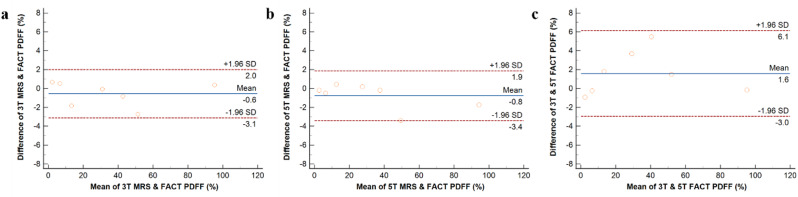
The Bland-Altman analysis for 3 T MRS and FACT-PDFF (**a**), 5 T MRS- and FACT-PDFF (**b**), and 3 T and 5 T FACT-PDFF (**c**) from phantom scans. Blue lines depict the mean differences (bias) of all measurements, and red dotted lines represent the ± 1.96 standard deviations. MRS, magnetic resonance spectroscopy; FACT, Fat Analysis and Calculation Technique; PDFF, proton density fat fraction

There was a strong linear correlation between FACT-PDFF and MRS-PDFF at 3 T and 5 T, with an R^2^ of 0.998 and 0.999 and an intercept of 0.434 and − 0.043, respectively. Additionally, there was a strong linear correlation between FACT-PDFF at 3 T and 5 T, with an R^2^ of 0.995 and an intercept of 1.514. In linear regression analyses of PDFF derived from different techniques and magnetic field strengths, all linear coefficients showed statistical significance while intercepts did not (Table S3). In the Bland-Altman analysis, all samples were located within the 95% CIs, suggesting that the deviations between MRS-PDFF and FACT-PDFF at 3 T and 5 T, as well as FACT-PDFF at different field strengths were small (Fig. 3).

### Repeatability of FACT-PDFF

**Fig. 4:**
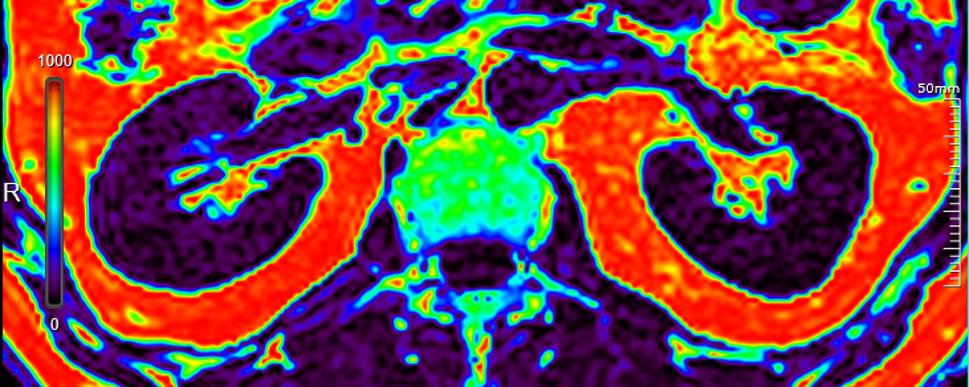
FACT-PDFF mapping at 5 T. The mean PDFF values (‰) of bilateral cortex, medulla, sinus fat and perirenal fat were 55.17, 49.67, 914.27, 975.33 (right kidney) and 37.17, 36.07, 928.13, 965.60 (left kidney), separately. FACT, Fat Analysis and Calculation Technique; PDFF, proton density fat fraction

In clinical study, there was no statistically significant difference in the image evaluation between the two radiologists (Z = 0.816, *P* = 0.414). The FACT images demonstrated high quality (all scores ≥ 4, Fig. 4). The ICCs for FACT-PDFF at all ROIs across three consecutive scans were in excellent agreement (0.837–0.991, Table 3). The CVs indicated that the variability of FACT-PDFF in bilateral cortex, medulla, renal sinus fat and perirenal fat across repeated scans were small (0.78%-4.49%, Table 3).

**Table 3:** Mean ICCs [means (95% CIs)] and CVs [means (95% CIs)] for FACT-PDFF between three repeated scan sessions

		ICC	CV (%)
Cortex	Left	0.901 (0.816–0.952)	4.49 (3.31–5.68)
	Right	0.949 (0.904–0.976)	2.71 (1.96–3.46)
Medulla	Left	0.867 (0.759–0.935)	5.17 (4.02–6.32)
	Right	0.913 (0.839–0.958)	3.23 (2.31–4.14)
Sinus fat	Left	0.958 (0.920–0.980)	2.34 (1.35–3.33)
	Right	0.991 (0.982–0.996)	2.10 (1.48–2.71)
Perirenal fat	Left	0.837 (0.710–0.919)	0.78 (0.53–1.02)
	Right	0.878 (0.778–0.940)	0.78 (0.54–1.02)

ICC, intraclass correlation coefficient; CI, confidence interval; CV, coefficient of variation; FACT, Fat Analysis and Calculation Technique; PDFF, proton density fat fraction

### Correlations between clinical features and FACT-PDFF

FACT-PDFF in left medulla was found positively correlated with age (*r* = 0.451, *P* = 0.027). FACT-PDFF in right cortex was found positively correlated with height (*r* = 0.425, *P* = 0.038). FACT-PDFF in right cortex, bilateral medulla and left sinus fat were found positively correlated with weight (*r* range, 0.513–0.784; *P* ≤ 0.010 for all). FACT-PDFF in right cortex, bilateral medulla, left sinus, and left perirenal areas were found positively correlated with waist (*r* range, 0.510–0.778; *P* ≤ 0.023 for all). FACT-PDFF in right cortex and bilateral medulla were found positively correlated with hip (*r* range, 0.665–0.760; *P* < 0.001 for all). FACT-PDFF in bilateral cortex, bilateral medulla, left sinus, and left perirenal areas were found positively correlated with BMI (r range, 0.412–0.750; *P* ≤ 0.046 for all). FACT-PDFF in right cortex, left medulla, left sinus fat and left perirenal areas were found positively correlated with waist-to-hip ratio (*r* range, 0.445–0.638; *P* ≤ 0.026 for all). FACT-PDFF in right cortex, bilateral medulla, and left perirenal areas were found positively correlated with waist-to-height ratio (*r* range, 0.608–0.797; *P* ≤ 0.002 for all). The correlations between clinical features and FACT-PDFF are listed in Table 4.

**Table 4:** Correlations between clinical features and FACT-PDFF

		Age		Height		Weight		Waist		Hip		BMI			Waist-to-hip ratio			Waist-to-height ratio	
		*r*	*P*	*r*	*P*	*r*	*P*	*r*	*P*	*r*	*P*	*r*	*P*	*r*		*P*	*r*		*P*
Cortex	Left	0.221	0.299	0.240	0.259	0.401	0.052	0.350	0.094	0.397	0.054	0.412	0.046	0.211		0.323	0.353		0.091
	Right	0.402	0.051	0.425	0.038	0.784	<0.001	0.749	<0.001	0.760	<0.001	0.750	<0.001	0.472		0.020	0.660		<0.001
Medulla	Left	0.451	0.027	0.261	0.217	0.662	<0.001	0.778	<0.001	0.670	<0.001	0.737	<0.001	0.602		0.002	0.797		<0.001
	Right	0.397	0.055	0.283	0.180	0.644	0.001	0.629	0.001	0.665	<0.001	0.659	<0.001	0.382		0.066	0.625		0.001
Sinus fat	Left	0.187	0.381	0.287	0.173	0.513	0.010	0.461	0.023	0.279	0.186	0.420	0.041	0.455		0.026	0.392		0.058
	Right	0.315	0.133	0.174	0.417	0.399	0.054	0.355	0.089	0.278	0.188	0.306	0.146	0.343		0.101	0.331		0.114
Perirenal fat	Left	0.316	0.133	-0.007	0.976	0.339	0.105	0.510	0.011	0.218	0.305	0.522	0.009	0.638		0.001	0.608		0.002
	Right	-0.102	0.637	0.003	0.989	0.040	0.853	0.022	0.920	-0.082	0.704	0.081	0.707	0.184		0.389	0.031		0.885

BMI: body mass index

### Comparisons of FACT-PDFF between bilateral kidneys and cortex/medulla fat

The averaged values of FACT-PDFF at each ROI from repeated scans are summarized in Table 5. In cortex and medulla, the FACT-PDFF of the left kidney was significantly lower than that of the right kidney (*P* < 0.001). The FACT-PDFF of the left renal sinus was significantly higher than that of the other side (*P* = 0.003). No statistically significant difference was observed between bilateral perirenal fat (*P* = 0.111).

The FACT-PDFF of renal cortex was significantly higher than that of renal medulla (42.80‰ vs. 36.94‰, *P* < 0.001).

**Table 5:** Comparison of mean FACT-PDFF (‰) obtained from repeated scans between bilateral kidneys

	Left	Right	*P*
Cortex	37.98 ± 6.24	47.61 ± 7.02	< 0.001
Medulla	30.55 ± 4.68	43.33 ± 5.30	< 0.001
Sinus fat	876.64 ± 110.67	787.45 ± 198.18	0.003
Perirenal fat	949.00 ± 21.35	957.42 ± 24.62	0.111

FACT, Fat Analysis and Calculation Technique; PDFF, proton density fat fraction

### Gender differences in renal and perirenal FACT-PDFF

In comparison of FACT-PDFF between genders, there were significant differences between male and female in right renal cortex, left renal medulla and left perirenal fat (*P* range, 0.006–0.044, Table 6).

**Table 6:** Gender differences in the mean of renal FACT-PDFF (‰) obtained from repeated scans

		Male	Female	*P*
Cortex	Left	39.45 ± 6.44	36.25 ± 5.81	0.530
	Right	50.51 ± 6.76	44.17 ± 5.88	0.010
Medulla	Left	32.16 ± 5.34	28.65 ± 2.98	0.006
	Right	44.67 ± 5.07	41.75 ± 5.34	0.177
Sinus fat	Left	942.44 ± 26.55	820.13 ± 144.36	0.514
	Right	861.43 ± 129.40	700.00 ± 233.91	0.675
Perirenal fat	Left	950.48 ± 18.12	947.25 ± 25.46	0.044
	Right	964.24 ± 15.54	949.36 ± 31.18	0.624

FACT, Fat Analysis and Calculation Technique; PDFF, proton density fat fraction

## Discussion

Our study comprehensively applied the FACT to quantify renal and perirenal fat, yielding results that offer valuable insights into renal fat distribution and its associations with various factors. We validated the feasibility of FACT-PDFF assessment at 5 T.

Although MRS was considered the reference method for fat quantification, especially in hepatic fat content quantification, recent advancements in 3 T FACT-PDFF have significantly improved its accuracy, making it an important method for quantifying fat content in clinical settings [23–26]. This study showed strong linear correlations between FACT-PDFF and MRS-PDFF at both 3 T and 5 T, validating FACT’s accuracy in measuring renal and perirenal fat fractions—consistent with previous studies demonstrating strong linear correlations between CSE-MRI-derived PDFF (1.5 T/3 T) and MRS-PDFF, which was similar to findings of our study [27, 28]. This indicated that FACT could be a reliable alternative to MRS in renal and perirenal region, especially considering its potential advantages in dealing with complex in-vivo environments.

The low bias of Bland-Altman plot and near-perfect correlations (R² ≥ 0.998) between FACT-PDFF and MRS-PDFF at both 3 T and 5 T indicated that FACT-PDFF achieved equivalent precision to MRS for fat quantification. This finding aligned with prior studies of hepatic diseases demonstrating the interchangeability of FACT-PDFF and MRS [14, 29]. As compared to corresponding MRS-PDFF at 3 T and 5 T, intercept values observed in our study (5 T: -0.043; 3 T: 0.403) suggested a reduction at the higher field. While without showing statistical significance, the closer-to-zero intercept at 5 T might be attributed to SNR dependent spectral fitting accuracy and the enhanced disentanglement of fat and water proton signals. This suggested that the precision of FACT-PDFF could be further optimized in the context of ultra-high field systems.

The excellent ICCs and CVs for FACT-PDFF across different anatomical positions demonstrated the remarkable repeatability of the FACT. In long-term clinical follow-up studies, this consistency was crucial. Current evidence suggested that renal fat deposition might play an important role in kidney damage [30]. Moreover, the qualitative assessment suggested that all FACT images in this study achieved good quality.

The differences in FACT-PDFF between the left and right kidneys in the cortex, medulla, and renal sinus were intriguing. Anatomical differences, such as variations in blood vessel distribution and innervation, might contribute to these disparities. Previous study indicated the investigators quantified renal sinus adipose tissue in both renal units, documenting asymmetric accumulation patterns, which might be attributed to inherent anatomical and functional disparities between the left and right kidneys [31]. The left and right kidneys might receive different blood flow patterns, which could affect fat metabolism and deposition. Hormonal regulation might also play a role, as the kidneys were under the influence of various systemic hormones. Statistically significant difference was detected in the mean FACT-PDFF values between the renal cortex and medulla, with the former demonstrating a higher mean value, while a study of renal fat fraction at 3 T claimed that PDFF from multi-echo Dixon-like imaging could not differentiate between renal cortex and medulla [32]. Relative to 3 T systems, 5 T MRI demonstrated superior SNR when the same spatial resolution was applied, or allows higher resolution imaging when SNR was maintained since the signal intensity is proportional to B0 field strength. Therefore, FACT imaging at 5 T would enable the detection of minute anatomical discrepancies within the kidney. Such advancements hold significant potential for enhancing the characterization of organ-specific physiological and pathological mechanisms.

The observed correlations between clinical characteristics and FACT-PDFF provided important clues about the factors influencing renal fat accumulation. Forbes SC, et al. indicated that aging was associated with an increase in fat mass which increases the risk for disease, morbidity and premature mortality [33]. In our present study, the finding of a positive correlation between age and left medullary FACT-PDFF (*r* = 0.451, *P* = 0.027) may be attributed to the small sample size and narrow age range (22 to 46 years). Future studies with larger cohorts were needed to confirm these relationships. Additionally, exploring the potential interactions between different clinical factors and their combined effects on renal fat distribution could further enhance our understanding of the underlying mechanisms.

The gender-based differences in FACT-PDFF were also significant. Sex hormones were known to regulate fat metabolism and distribution [34]. Testosterone in males and estrogen in females had distinct effects on adipocyte function and lipid storage [34, 35]. In addition to hormonal factors, lifestyle differences between genders, such as diet and physical activity levels, could contribute to the observed differences in renal fat distribution.

Compared to lower field strengths, several studies had shown the feasibility and improvement of abdominal imaging at 5 T. For structural imaging, images exhibited higher SNR and better visualization of small vessels and lesions at 5 T [36–41]. Besides, quantitative MRI parameters such as apparent diffusion coefficient derived from diffusion-weighted imaging, and FACT-PDFF at 5 T showed good repeatability and consistency with traditional field strengths [14, 36, 37, 42, 43]. However, as magnetic field strength increases, 5 T MRI faces challenges such as B0 and B1 field inhomogeneities [44]. At 5 T, increased chemical shift degrades B0 homogeneity; for B0 shimming in this study’s 5 T system, a pre-scan low-resolution dual-echo GRE sequence was used for B0 mapping, followed by active shim coils adjusting currents to generate a supplemental magnetic field—superimposed on the uncorrected field to calibrate B0. 5 T MRI also faces B1 inhomogeneity from shortened B1 wavelength due to elevated Larmor frequency, causing standing wave effects. For B1 shimming, the scanner integrated an 8-channel volume transmit coil with independently connected RF power amplifiers and uniform body distribution, which transmitted and adjusted RF in parallel to ensure homogeneous excitation over a large volume.

It is important to acknowledge the study limitations: the small sample size limits statistical power and generalizability, and the focus on healthy volunteers hinders exploring the clinical relevance of 5 T FACT-derived renal fat metrics in disease. Future research should include kidney disease patients, supplement missing covariates, and adopt coronal imaging to optimize tissue coverage.

## Conclusions

The study successfully demonstrated that the FACT technique can accurately quantify renal and perirenal fat at 5 T magnetic field strength, enhancing the understanding of renal fat distribution and its clinical associations and potentially advancing comprehensive research on the role of renal fat in related diseases to optimize diagnostic and therapeutic strategies.

## Supplementary Information

Below is the link to the electronic supplementary material.

Supplementary Material 1

## Data Availability

Data and materials will be provided upon reasonable request by the corresponding author.

## Abbreviations

ANCOVA: Analysis of Covariance
BMI: Body Mass Index
CSE-MRI: Chemical Shift-Encoded MRI
CV: Coefficient of Variation
FACT: Fat Analysis and Calculation Technique
FOV: Field of View
ICC: Intraclass Correlation Coefficient
MRI: Magnetic Resonance Imaging
MRS: Magnetic Resonance Spectroscopy
PDFF: Proton Density Fat Fraction
PRESS: Point-Resolved Spectroscopy
RF: Radio Frequency
ROI: Region of Interest
SNR: Signal-to-Noise Ratio
STEAM: Stimulated Echo Acquisition Mode
TE: Echo Time
TR: Repetition Time
