# Supplementary material for: Quantitative assessment of renal and perirenal adipose tissue distribution at 5 T: a feasibility study
Source: BMC Med Imaging. 2025 Dec 29;26:59. doi: 10.1186/s12880-025-02136-8 (PMC12859936; doi:10.1186/s12880-025-02136-8)
Supplement: Supplementary file 1 — Supplementary Material 1 [file 12880_2025_2136_MOESM1_ESM.docx]

**Supplementary Tables**

**Table S1** Kolmogorov-Smirnov normality tests for continuous data in volunteer study (n=24). Normal distribution is rejected with P <0.05

| Category | P values |
| --- | --- |
| Age | <0.001 |
| Height | 0.200 |
| Weight | 0.066 |
| Waist | 0.200 |
| Hip | 0.200 |
| Body mass index | 0.084 |
| Waist-to-hip ratio | 0.091 |
| Waist-to-height ratio | 0.200 |
| Right cortex FACT-PDFF | 0.006 |
| Right medulla FACT-PDFF | 0.050 |
| Right sinus FACT-PDFF | <0.001 |
| Right perirenal FACT-PDFF | 0.200 |
| Left cortex FACT-PDFF | 0.200 |
| Left medulla FACT-PDFF | 0.013 |
| Left sinus FACT-PDFF | <0.001 |
| Left perirenal FACT-PDFF | 0.200 |

FACT, Fat Analysis and Calculation Technique; PDFF, proton density fat fraction

**Table S2** Post-hoc power analyses in volunteer study (n=24)

| Tests | ROI | Power |
| --- | --- | --- |
| Paired samples t-test comparing FACT-PDFF of bilateral kidneys | Cortex | >0.999 |
|  | Medulla | >0.999 |
|  | Sinus fat | 0.885 |
|  | Perirenal fat | 0.348 |
| Paired samples t-test comparing FACT-PDFF of cortex and medulla | Cortex vs. medulla | >0.999 |
| Comparisons of FACT-PDFF between sex | Right cortex | 0.655 |
|  | Right medulla | <0.001 |
|  | Right sinus fat | 0.490 |
|  | Right perirenal fat | <0.001 |
|  | Left cortex | <0.001 |
|  | Left medulla | 0.490 |
|  | Left sinus fat | 0.618 |
|  | Left perirenal fat | <0.001 |

The post-hoc power was calculated by setting P at 0.05. ROI, region-of-interest; FACT, Fat Analysis and Calculation Technique; PDFF, proton density fat fraction

**Table S3** Statistical significances in linear regression analyses of PDFF from different techniques and magnetic field strengths in phantom study (n=7)

| Variables | P for linear coefficient | P for intercept |
| --- | --- | --- |
| 3 T MRS-PDFF & 3 T FACT-PDFF | <0.001 | 0.618 |
| 3 T MRS-PDFF & 5 T FACT-PDFF | <0.001 | 0.527 |
| 5 T MRS-PDFF & 3 T FACT-PDFF | <0.001 | 0.342 |
| 5 T MRS-PDFF & 5 T FACT-PDFF | <0.001 | 0.951 |
| 3 T & 5 T FACT-PDFF | <0.001 | 0.340 |

PDFF, proton density fat fraction; MRS, magnetic resonance spectroscopy; FACT, Fat Analysis and Calculation Technique

**Fat-water phantom construction**

A universal fat-water phantom was constructed containing 7 cylinders with a range of the volume fraction of the fat (0%, 10%, 20%, 30%, 40%, 50%, and 100%) as described by Hines et al [1] and Schneider [2], The “fat” component was organic peanut oil (0.916 g/cm3). The “water” component comprising the water fraction of the phantom contained the following in distilled deionized water: 43 mM sodium dodecyl sulfate, 43 mM sodium chloride, 3.75 mM sodium azide, and 0.3 mM gadobenate dimeglumine. Agar (2.0% w/v) was added over heat with stirring util melted. Volumes of the water solution were poured into vials containing premeasured peanut oil, mixed through gentle inversion for approximately 2 min, and formed a solid gel when cooled to room temperature.

**References**

[1] Hines, Catherine D G et al. “T1 independent, T2* corrected MRI with accurate spectral modeling for quantification of fat: validation in a fat-water-SPIO phantom.” Journal of magnetic resonance imaging: JMRI vol. 30,5 (2009): 1215-22. doi:10.1002/jmri.21957

[2] Schneider, Erika et al. “Long-term inter-platform reproducibility, bias, and linearity of commercial PDFF MRI methods for fat quantification: a multi-center, multi-vendor phantom study.” European radiology vol. 31,10 (2021): 7566-7574. doi:10.1007/s00330-021-07851-8
